# Imaging as a Personalized Biomarker for Prostate Cancer Risk Stratification

**DOI:** 10.3390/diagnostics8040080

**Published:** 2018-11-30

**Authors:** Kyle H. Gennaro, Kristin K. Porter, Jennifer B. Gordetsky, Samuel J. Galgano, Soroush Rais-Bahrami

**Affiliations:** 1 Department of Urology, University of Alabama at Birmingham, Birmingham, AL 35294, USA; kgennaro@uabmc.edu (K.H.G.); jgordetsky@uabmc.edu (J.B.G.); 2 Department of Radiology, University of Alabama at Birmingham, Birmingham, AL 35294, USA; kkporter@uabmc.edu (K.K.P.); samuelgalgano@uabmc.edu (S.J.G.); 3 Department of Pathology, University of Alabama at Birmingham, Birmingham, AL 35294, USA

**Keywords:** magnetic resonance imaging (MRI), Gleason score, cancer staging, active surveillance, radical prostatectomy, radiation therapy

## Abstract

Biomarkers provide objective data to guide clinicians in disease management. Prostate-specific antigen serves as a biomarker for screening of prostate cancer but has come under scrutiny for detection of clinically indolent disease. Multiple imaging techniques demonstrate promising results for diagnosing, staging, and determining definitive management of prostate cancer. One such modality, multiparametric magnetic resonance imaging (mpMRI), detects more clinically significant disease while missing lower volume and clinically insignificant disease. It also provides valuable information regarding tumor characteristics such as location and extraprostatic extension to guide surgical planning. Information from mpMRI may also help patients avoid unnecessary biopsies in the future. It can also be incorporated into targeted biopsies as well as following patients on active surveillance. Other novel techniques have also been developed to detect metastatic disease with advantages over traditional computer tomography and magnetic resonance imaging, which primarily rely on defined size criteria. These new techniques take advantage of underlying biological changes in prostate cancer tissue to identify metastatic disease. The purpose of this review is to present literature on imaging as a personalized biomarker for prostate cancer risk stratification.

## 1. Introduction

The National Institutes of Health Biomarkers Definitions Working Group defines a biomarker as “a characteristic that is objectively measured and evaluated as an indicator of normal biological processes, pathogenic processes, or pharmacologic responses to a therapeutic intervention” [1]. Historically, prostate-specific antigen (PSA) has been used as a biomarker for prostate cancer screening. PSA combined with physical exam findings, including a digital rectal exam (DRE), would indicate the need for further evaluation with a systematic biopsy [2]. However, the widespread adoption of PSA screening for prostate cancer in the 1990s led to a significant increase in the incidence of prostate cancer [3]. This increase in incidence is largely attributable to increased detection of low-risk disease that may not affect patient mortality. Consequently, the United States Preventative Services Task Force decreased the grade of recommended prostate cancer screening and a significant decrease in the number of biopsies has since followed. Since then, there have been fewer lower-risk prostate cancers diagnosed but also a proportionally fewer number of intermediate- and high-risk prostate cancers found based on the decreased widespread PSA-based prostate cancer screening [4]. For these reasons, improved biomarkers for prostate cancer are needed, specifically for the diagnosis of more clinically significant cases where early detection and definitive treatment would be most impactful in decreasing disease-specific morbidities and mortality.

As the data continues to evolve, imaging is developing a growing role as a personalized biomarker to guide diagnosis of prostate cancer, safe patient selection for enrollment in active surveillance (AS) protocols, and optimized definitive management options and techniques. Multiparametric magnetic resonance imaging (mpMRI) has been at the forefront of these developments and provides excellent anatomical and functional imaging of the prostate as well as increased detection of clinically significant disease and decreased sensitivity for clinically insignificant disease [5]. mpMRI has been shown to accurately define local prostate cancer involvement as well detection of regional and metastatic disease. Additionally, multiple novel imaging techniques have been developed for the purpose of detecting metastatic disease with promising results. These developments continue to shape the future role imaging will play in prostate cancer risk stratification. Herein, we aim to review the present literature on imaging as a personalized biomarker for prostate cancer risk stratification.

## 2. Materials and Methods

A literature search of Pubmed was conducted using the following keywords in combinations: “prostate cancer”, “imaging”, “risk stratification”, “multiparametric MRI”, “active surveillance”, “lymphography”, “diffusion weighted imaging”, “staging”, “MRI targeted biopsy”. Only English language clinical studies, clinical trials, comparative studies, multicenter trials, randomized control trials, and validation studies published between 1 January 2015 and 15 July 2018 accessible through our institution were included and were reviewed for applicability to the review topic. The bibliographies of the identified studies were used to expand the search.

## 3. Role of Multiparametric MRI in Prostate Cancer Diagnosis and Local Staging

mpMRI includes high-resolution T2-weighted sequences (T2), diffusion-weighted sequences (DWI), and dynamic contrast-enhanced images (DCE) [6]. mpMRI has demonstrated a great sensitivity in the detection of prostate cancer compared to standard techniques and correlates well with findings at final pathology [7,8]. Some groups have developed imaging models and computer-aided diagnosis systems incorporating mpMRI with promising results [9,10]. For these reasons, the role of mpMRI continues to grow in the initial work up and evaluation of prostate cancer [11].

In an attempt to standardize imaging findings, the Prostate Imaging Reporting and Data System (PIRADS) was developed in 2012. PIRADS quantified lesions found on mpMRI on a scoring scale from 1 to 5 based on the likelihood of the presence of disease [12]. This was subsequently revised in 2015 with the development of PIRADS v2 in order to decrease variability between interpretations. Studies have since shown similar diagnostic accuracy between the two versions of PIRADS with improved inter-reader agreeability for PIRADS v2 [13,14,15,16,17]. There is a substantial body of evidence that mpMRI positively correlates with the presence of underlying disease. In addition, there is an increased likelihood of detecting clinically significant prostate cancer with increasing PIRADS score [6,8,14,16,18,19,20,21,22,23,24,25,26,27,28,29,30]. Inverse findings hold true as well, in that a PIRADS score of ≤2 has a good negative predictive value (NPV) for excluding any cancer and even better for excluding clinically significant prostate cancers (Gleason scores of 7–10, Grade Groups 2–5) [21]. Furthermore, cancers missed on mpMRI are often low volume and clinically insignificant [22]. These findings could potentially impact the selection of patients who will require a prostate biopsy and provide a more selective process for determining which patients undergo an invasive diagnostic procedure with a goal of early detection of more clinically significant cases [31].

When incorporating additional data, the detection rate of prostate cancer by mpMRI is even greater as demonstrated by Dinh et al., who implemented a model that included prevalence maps and patient biopsy results to improve rates of cancer detection [32]. Patient-specific factors can also be included, such as DRE findings and PSA, to improve the sensitivity and specificity of mpMRI findings. In a study by Thompson et al., the authors showed a NPV of 90% in men with a negative mpMRI, normal DRE, and PSA < 10 ng/mL, which could save 27% of men in their study an unnecessary biopsy [28]. However, they did note that mpMRI would have missed six significant prostate cancer diagnoses in their study population of 334 men. Other groups have found similar findings and suggest that biopsy could be avoided in men with negative mpMRI [33] and that mpMRI may even one day be used as a primary screening test for prostate cancer [16].

Preoperative imaging findings can also impact surgical planning based on tumor location, extraprostatic extension (EPE), and suspected lymph node metastasis. Multiple studies have demonstrated excellent concordance between tumor volume and anatomical location of lesions seen on mpMRI with findings on biopsy and radical prostatectomy [28,34,35]. mpMRI had a sensitivity of over 80% for detecting apical prostate cancer, which could guide surgical planning to maximize the amount of membranous urethral length preserved in order to improve incontinence [36]. Detection of these apical tumors is especially important given that standard biopsy alone is not a significant predictor of apical involvement [36]. In terms of lymph node involvement, mpMRI has been shown to have good sensitivity and specificity for the detection lymph node metastasis with 55–93% and 90–96%, respectively [37,38].

mpMRI can also detect EPE on staging imaging; this impacts disease management and utilization of nerve-sparing approaches with improved patient counseling [39,40,41,42]. The sensitivity and specificity for mpMRI detection of EPE is as high as 74% and 94%, respectively [43,44]. Even when definite EPE is not visualized on MRI, suspicious findings such as irregularity of the capsule, bulging/loss of capsule, and neurovascular bundle thickening can be used in conjunction with established nomograms (such as Partin tables and MSK nomogram) to more accurately predict EPE on final pathology [40]. Nomograms incorporating DWI have an improved sensitivity for detecting EPE with specificity as high as 100% [45]. The greatest advantage of mpMRI over established nomograms for predicting EPE is that mpMRI can localize the site of EPE, aiding in surgical planning and dissection and helping to avoid positive margins [40,43].

## 4. MRI-Targeted Prostate Biopsy

The current standard practice for prostate biopsy involves a systematic approach, taking samples from different regions of the prostate [2]. The biggest disadvantage of this technique is the systematic but essentially random approach to diagnosis. Incorporating mpMRI findings into biopsy practices allows for a focused biopsy of an area of interest and, in theory, detection of more clinically significant disease.

The incorporation of mpMRI with biopsy techniques can be done using multiple methods including cognitive fusion, in-bore biopsy, and fusion biopsy [46]. With cognitive fusion, the MRI data, including areas of suspicion, are known to the operator and additional cores are taken from the area of interest [47]. In-bore biopsy is a direct MRI-guided biopsy, and fusion-guided biopsy combines MRI with real-time ultrasound (US) guidance [47,48]. Some studies have demonstrated a significantly higher detection rate with MRI/US-fusion biopsy over cognitive-fusion biopsy when comparing cancer detection rates between the two techniques [49,50,51]. However, other groups have shown different results [52]. Taking both sagittal and axial MRI-guided cores during MRI-guided techniques has been shown to improve detection [53]. Although it is highly debated, higher rates of detection have been shown, particularly in the anterior zone, using a cognitive-fusion transperineal approach versus transrectal MRI/US-fusion-targeted biopsy in men with a previous negative systematic biopsy [54].

In comparison to standard biopsy, MRI-guided biopsy studies have demonstrated similar rates of cancer detection in both biopsy naïve patients and in patients with previous negative biopsies [5,47,48,55,56,57,58]. However, higher rates of detection with MRI-guided biopsies have also been shown [19,20,58,59,60,61,62]. In patients with previous negative biopsies, studies have demonstrated cancer detection rates up to 50% with MRI-guided biopsy, with approximately 30% representing clinically significant disease [19,21,63,64]. On average, MRI-guided biopsy also requires fewer cores and, as would be expected, has a significantly higher cancer detection rate per core [19,31,59,65,66,67,68,69,70,71,72].

Despite these findings, it must be noted that studies have demonstrated clinically significant prostate cancer that would otherwise be detected on standard or saturation biopsy will be missed on targeted biopsy at rates ranging from 2–25% [5,20,25,48,54,55,66,67,69,72,73,74]. Thus, the standard biopsy cannot yet be entirely replaced by targeted biopsy based on the current data. Many studies have demonstrated that a combination of both targeted biopsy and systematic biopsy yields the highest rate of detection in both biopsy naïve men and those on AS [19,21,69,71,72,73]. However, in detecting these additional clinically significant cancers, a substantial number of clinically insignificant cancers will also be detected [5].

Gleason score at the time of biopsy can have a tremendous impact on management decisions, such as whether a patient is appropriate for AS, whether a nerve-sparing approach is required or if a pelvic lymph node dissection is needed. For these reasons, an accurate diagnosis on biopsy is of utmost importance. It is known that Gleason score is frequently upgraded between initial biopsy and final pathology results, with an upgrade rate as high as 50% with standard biopsy [65]. In theory, MRI-guided biopsies should reduce the risk of upgrading at time of final pathology, since mpMRI can help the operator identify the most significant disease for biopsy. Arsov et al. demonstrated a trend towards reduced upgrading on MRI-guided biopsy when compared to standard biopsy, but the difference was not significantly different (40% vs. 50%) [65] Other groups have shown similar results (33% vs. 44%) [75]. However, when the two biopsy methods were used in combination, standard and MRI-guided biopsy, the risk of upgrading on final pathology substantially decreased from 28.8% to 18% [65,75]. Using the two methods in combination provides the best prognostic information for the patient and the most accurate results to guide further care. However, ongoing investigations are underway to assess the safety and efficacy of MRI target-based sampling in isolation without systematic sampling in specific patient populations.

One key difference between standard and MRI-guided biopsy is the rate at which clinically significant cancer is detected. Multiple studies have shown that targeted biopsies detect a greater number of clinically significant prostate cancers and fewer insignificant prostate cancers [5,20,25,26,50,57,62,66,68,69,76]. These results have been seen in both biopsy naïve men as well as men with previous negative biopsies [25,26,50,57]. Notably, this effect is more apparent in men with larger prostates (>50 cc) [61]. Reducing detection of clinically insignificant prostate cancer would decrease the number of men enrolled in AS and the associated costs of following this patient population.

Kasivisvanathan et al. recently published a noninferiority trial in which 500 men were randomized to either standard biopsy or mpMRI and MRI-guided biopsy, if indicated. In the MRI group, 28% of men were found to have negative mpMRI (PIRADS 2 or less) and did not undergo biopsies. In agreement with other published data, they showed a higher detection rate of clinically significant prostate cancer (Gleason score 3 + 4 or higher) in the MRI-guided group in comparison to standard biopsy (38% vs. 26%). There were also fewer men diagnosed with clinically insignificant cancer (9% vs. 22%). They did note similar rates of upgrading at time of prostatectomy—17% with MRI-guided biopsy versus 15% with standard biopsy. They also found that 16% of men who initially underwent a standard biopsy underwent further detection workup compared to just 3% in the MRI-guided biopsy group. From these results, they concluded that the MRI ± biopsy was superior to the standard biopsy with similar side effects [31].

Another additional benefit of targeted biopsy is that when patients are deemed appropriate for AS based on these results, clinicians and patients may have increased confidence in this decision [67]. Patients deemed appropriate for AS based on standard biopsy might be upgraded on a following targeted biopsy [63]. There is a correlation between the suspicion score of MRI lesions (using PIRADS v2) and the frequency of Gleason score upgrading with targeted biopsy when compared to concurrent systematic biopsy or to prior biopsy with systematic sampling alone [72]. Notably, it has been shown that patients initially diagnosed by MRI/TRUS-fusion biopsy have a significantly elevated chance of continuing on AS compared to those diagnosed by standard biopsy (80% vs. 50%) [76,77]. Additionally, patients with mpMRI and targeted biopsy have been shown to select AS with more confidence than a contemporary cohort of men undergoing systematic biopsy only, despite counseling by the same urologists [77].

In addition to Gleason score, other factors such as perineural invasion (PNI) are detected more effectively on targeted biopsy and can have significant clinical implications [78]. Detection of PNI is significantly associated with EPE and early biochemical recurrence (BCR). Detection of PNI on MRI-targeted biopsy has shown a sensitivity and specificity of 40% and 81% for EPE, suggesting that nerve-sparing approaches may not be appropriate in these patients [79].

## 5. Role of Multiparametric MRI in Active Surveillance

Active surveillance is commonly used to follow patients with low risk, indolent prostate cancer. One of the greatest limitations of AS is the need for repeat biopsies, which expose patients to potential complications related to the procedure [80]. mpMRI may play a significant role in following this subset of patients and guide clinicians in selecting patients who may need additional biopsies [55,81]. Alberts et al. demonstrated that patients with a PSA density of <0.15 ng/mL^2^ and MRI findings of PIRADS 1–3 showed no Gleason score upgrading on both standard and targeted biopsy, thus arguing that these patients might not benefit from additional biopsies [55]. Similarly, Radtke et al. demonstrated that a negative mpMRI had a NPV of 93.5%, thereby allowing for subsequent disqualification from AS [76]. This would limit the number of biopsies performed on patients on AS and reduce exposure to the risks involved with biopsy. Other groups have shown comparable results in patients with previous negative biopsies [21].

In a recent study by Dianat et al., men on AS underwent mpMRI and standard biopsy. In comparing patients with visible tumor on mpMRI to those with tumors not visible on mpMRI, there was less adverse pathology (clinically significant disease) detected on patients with MR-invisible tumors (8.3% vs. 40.5%) [82]. The authors argued that given the prognostic significance of MR-invisible tumors, the frequency of surveillance biopsies might be tailored in this population [82]. Other risk stratification nomograms have been developed for safe selection of patients diagnosed with low-risk prostate cancer who have undergone mpMRI and targeted biopsy [83,84]. These tools incorporating data from MRI findings may ultimately aid in selection of optimized biopsy techniques and intervals between biopsy sessions for men seeking ongoing confirmation for safe AS eligibility and continuation.

A retrospective study using the Korean Prostate Cancer database looked at patients with low-risk prostate cancer that would have been appropriate for AS, who had an mpMRI and eventually underwent radical prostatectomy. They analyzed the role of mpMRI in predicting patients who had upstaging or upgrading of their prostate cancer on subsequent prostatectomy. The authors found that models incorporating mpMRI significantly improved the diagnostic accuracy for predicting clinically significant prostate cancer and provided valuable information in this patient population [81].

There is also noted to be a correlation between PIRADS score and the presence of clinically significant disease in patients on AS [47,76]. Radtke et al. showed a 62.5% chance of harboring a Gleason score of 3 + 4 or greater disease in PIRADS 5 lesions. They also noted that an increase in the PIRADS score on consecutive MRI was a significant predictor of disqualification from AS and could serve as an indicator for the need for repeat biopsy [76]. It is important to note that 5 alpha-reductase inhibitors, such as Finasteride, may affect the interpretation of DWI imaging and the radiologist should be made aware if the patient is on this treatment. Nevertheless, mpMRI and MRI-targeted biopsy has been shown to be fruitful even in patients actively managed with 5 alpha-reductase inhibitors [85,86]. However, clinicians should have a lower threshold for triggering biopsy in patients on Finasteride [87].

## 6. Role of Imaging in Regional Staging and Evaluation for Metastatic Lymph Node Involvement

Accurate risk assessment is critical for determining further interventions following a diagnosis of prostate cancer on biopsy, especially given the heterogeneity of prostate cancer. Risk assessment tools are able to predict lymph node involvement based on the patient’s age, PSA, Gleason score, percent of positive cores, and pathologic stage [88,89,90,91]. According to the National Comprehensive Cancer Network Practice Guidelines in Oncology: Prostate Cancer version 2.2018, patients with a risk of lymph node involvement greater than 10% are recommended to undergo further staging imaging [92]. Current staging imaging includes either a computed tomography (CT) or MRI of the pelvis and abdomen combined with a nuclear medicine bone scan, based on the patient’s risk of metastatic disease [92]. CT and MRI rely primarily on size criteria to identify the suspected presence of nodal metastatic disease. This results in false-positive imaging findings in enlarged nodes with reactive hyperplasia and false-negative imaging in cases where smaller nodes harboring malignant cells are not recognized as sites of metastasis [93,94]. The sensitivity and specificity for CT imaging to identify nodal metastatic disease has been demonstrated to be 42% and 82%, respectively, with similar rates observed on MRI, with a reported sensitivity of 39% and a specificity of 82% [93]. For these reasons, much work has been done to develop imaging techniques that take advantage of the cellular biologic changes of malignant cells to identify disease spread to lymph nodes (Table 1).

One such technique utilizes ultrasmall super paramagnetic iron oxide (USPIO) to highlight malignant cells on MRI [95,96]. These iron particles are taken up by macrophages, which are transported to lymph nodes via the lymphatic vessels. Disturbances to lymphatic flow lead to decreased accumulation of these iron particles in malignant cells. Utilizing this strategy, lymph nodes harboring metastatic disease will retain their signal on T2 images while normal lymph nodes will demonstrate signal loss [96,97]. The advantage of using USPIO with MR lymphangiography (MRL) is detection of malignant nodes at smaller sizes and improved sensitivity in this size range. One of the first USPIOs used was ferumoxtran-10, which demonstrated a sensitivity of 96.4% for detecting occult disease in lymph nodes in the 5–10-mm range, compared to a sensitivity of 28.5% with MRI alone [96]. However, ferumoxtran-10 was not approved by the United States Food and Drug Administration (FDA) due to concerns about insufficient data to support a broad indication for use across all cancer types. Ferumoxytol, another USPIO that is FDA approved as an iron replacement therapy in patients with chronic kidney failure, has shown promising results but does have a weaker signal suppression compared to ferumoxtran-10 MRL [98]. One group of authors argued that clinicians could forego pelvic lymph node dissection in patients with negative studies using these agents [99]. However, further research is needed to validate their accuracy.

Another emerging technology for prostate cancer staging is the utilization of positron emission tomography (PET), which is a form of functional imaging using positron-emitting radiotracers combined with either CT or MRI for anatomic localization. The radiotracers used in staging and restaging prostate cancer are [^18^F]fluorodeoxyglucose (FDG), [^11^C]Choline, [^18^F]Choline, [^11^C]acetate, [^18^F]Sodium fluoride, and [^18^F]fluciclovine. The most commonly utilized PET radiotracer in imaging of patients with cancer is 2-deoxy-2-(^18^F)fluoro-d-glucose (FDG). However, it has limited use in prostate cancer due to the low metabolic activity of this disease in its early stage, which results in poor sensitivity [100]. Additionally, FDG is excreted in the urine, which may decrease the sensitivity of identifying pelvic lymph node metastasis [101].

[^18^F]sodium fluoride (NaF) has been used for the detection of osseous metastases in patients with prostate cancer. Uptake of NaF is related to bone turnover, and in areas of bone metastases where there is increased bone turnover, NaF will bind to newly deposited and mineralized bone. Studies have demonstrated superior sensitivity and specificity of NaF PET/CT for detection of osseous metastases (greater than 95%) when compared to conventional skeletal scintigraphy utilizing ^99m^Tc-methyldiphosphinate [102]. However, these radiotracers play no role in identifying lymph node metastases.

One of the FDA-approved radiotracers, [^11^C]Choline, uses a substrate of the cell membrane metabolic pathway, which is upregulated in prostate cancer cells [103]. This radiotracer is currently FDA approved for the detection and localization of disease in BCR but is not currently clinically useful for initial staging due to limited sensitivity [104,105]. Fluorinated analogues of choline, such as [^18^F]fluorocholine and 2-[^18^F]-Fluoroethylcholine, utilize the longer half-life of fluorine-18 [39,106]. However, studies have demonstrated similar sensitivities and specificities of all choline-based radiotracers for the detection of metastatic lymph nodes. In addition, these tracers underestimate the total amount of pelvic nodal metastases compared to findings on pelvic lymph node dissection [107]. Also, these fluorinated choline analogs are excreted in the urine, which could impair detection of pelvic lymph node disease [108]. [^18^F]-Choline, when used with PET/MRI, has shown to have a significantly higher sensitivity that mpMRI alone but no significant improvement in detection of seminal vesicle invasion or EPE [109,110]. Furthermore, ^18^F-Choline PET/MRI has been implemented into fusion biopsies with promising results [111].

Acetate-based radiotracers can be utilized in prostate cancer patients due to the overexpression of the fatty acid metabolic pathway [112,113]. An initial study demonstrated a sensitivity of 68% and specificity of 78.1% for detection of lymph node metastases in patients with intermediate- or high-risk prostate cancer undergoing radical prostatectomy [114]. In a subsequent study, [^11^C]acetate demonstrated a sensitivity of 38% and specificity of 96% in intermediate- and high-risk prostate cancer patients undergoing extended lymph node dissection which was lower than previous studies and attributed to rigid criteria requiring substantial uptake in lymph nodes for a positive diagnosis [115].

Amino acid radiotracers accumulate in prostate cancer cells through the upregulation of transmembrane amino acid transport. [^18^F]fluciclovine is FDA approved for use in BCR and has promising preliminary results with use in initial staging with similar sensitivity and specificity as conventional imaging with improved detection in 5–9-mm nodes and skeletal lesions not detected by conventional imaging [116]. An early clinical trial investigating the use of [^18^F]fluciclovine PET/MRI for preoperative lymph node staging demonstrated a sensitivity of 40% and specificity of 87.5% and lacked the sensitivity to replace pelvic lymph node dissection at time of prostatectomy [117]. Additional research regarding detection of localized prostate cancer demonstrated a significant correlation between quantitative [^18^F]fluciclovine PET maximum standardized uptake values and Gleason score but failed to outperform mpMRI in lesion detection [118]. However, mpMRI combined with fluciclovine PET demonstrate area under the curve above 90% and has potential for improving detection and characterization of high-risk prostate cancer when compared to mpMRI and PET alone [119]. Although not studied in the pretreatment staging setting, fluciclovine has been shown to be superior to choline radiotracers in patients with BCR [120].

Multiple new PET tracers have been developed that utilize prostate-specific membrane antigen (PSMA) as their target. Prostate cancer cells overexpress PSMA, which is a transmembrane protein, and different radiotracers have targeted both intracellular and extracellular components [121]. The earliest of these radiotracers is [^111^In]capromab pendetide, a radiolabeled monoclonal antibody that targets the intracellular portion of PSMA and is imaged utilizing SPECT/CT. Although there is some increase in detection of metastatic disease over conventional imaging, sensitivity and specificity are extremely limited, which hinders its potential benefit [122,123]. The targeting of the intracellular component of PSMA requires a delay of 5–7 days between injection and imaging and only allows for imaging in cells that have undergone apoptosis or necrosis [124]. For this reason, multiple radiotracers that target extracellular components have been developed and allow for imaging of viable cancer cells.

One such agent, *N*-{*N*-[(*S)*-1,3-dicarboxypropyl] carbamoyl},-4-(^18^F)fluorobenzyl-l-cysteine [(^18^F)DCFBC], binds irreversibly to the extracellular component of PSMA [125]. However, it has been shown to have a limited role in detecting localized prostate cancer except as an adjunct to mpMRI [126]. Another such agent, ^68^Ga-*N*,*N*;-bis [2-hydroxy-5-(carboxyethyl)benzyl] ethylenediamine-*N*,*N*′-diacetic acid ([^68^Ga]HBED-CC), has demonstrated sensitivity and specificity superior to other imaging techniques [127]. [^68^Ga]-HBED-CC PET/CT has been shown to improve accuracy of lymph node staging over conventional imaging in the setting of BCR with a sensitivity of 65.9% and specificity of 98.9% [128]. Also, [^68^Ga]-HBED-CC PET has been shown to be superior to traditional bone scintigraphy in determining metastatic status of patients, with favorable sensitivity and specificity [129]. Early work done with [^68^Ga]PSMA PET/CT has demonstrated a sensitivity of 33.3% and specificity of 100% for lymph node metastasis detection in primary staging of high-risk prostate cancer [130]. In comparison to other radiotracers, [^68^Ga]PSMA, has shown a higher rate of detection for metastatic lymph nodes with a greater effect in patients with a PSA < 1 for detection of BCR. Additionally, it was also noted to have higher tracer uptake in the prostate [108]. Similar to [^18^F]choline, use of [^68^Ga]PSMA may play a future role in targeted biopsy [131,132] as well as in radiation therapy [132]. Another cell membrane target that has demonstrated promising results is urokinase plasminogen activator receptor (uPAR). It shows limited activity in benign cells but is overexpressed in multiple malignancies including breast, bladder, and prostate cancer. A gallium-labeled peptide agonist for this receptor, AE105, has shown to be safe, well tolerated, and useful in the identification of primary tumors and metastases in phase I trials [133].

Although not currently the standard of care, PET imaging may play a role in the future for risk stratification and detection of metastatic disease in the pretreatment setting through staging imaging, evaluation for recurrent disease, and use in fusion biopsies. Additional radiotracers, including gastrin-related peptide receptor and testosterone analogues, are under development and further research will be needed to determine the optimal PET imaging agent for prostate cancer.

## 7. Conclusions

In conclusion, there is a growing body of evidence that supports the role of imaging as a personalized biomarker for prostate cancer risk stratification. mpMRI is at the forefront of these imaging techniques and can diagnose prostate cancer as well as accurately stage local disease. Combined with targeted biopsy, mpMRI has shown improved detection of clinically significant cancer while simultaneously limiting the diagnosis of clinically insignificant cancer. mpMRI may one day replace current diagnostic techniques. Additionally, it improves patient selection for AS and may limit unnecessary repeat biopsies in the AS population. Finally, there are also many PET radiotracers under investigation to improve the diagnosis of metastatic disease both at time of diagnosis and biochemical recurrence.

## Figures and Tables

**Table 1:** Imaging techniques targeting prostate cancer biology.

Target	Mechanism	Example Agents
USPIO (Ultrasmall paramagnetic iron)	Uptake by macrophages with decreased accumulation in malignant cells due to alterations in lymphatic flow	Ferumoxtran-10 Ferumoxytol
Sodium fluoride	Radiotracer; uptake in areas of increased bone turn over	[^18^F]Sodium fluoride
Choline	Radiotracer; substrate of cell membrane metabolic pathway that is upregulated in prostate cancer	[^11^C]Choline [^18^F]fluorocholine 2-[^18^F]-Fluoroethylcholine
Acetate	Radiotracer; increased uptake due to overexpression of fatty acid metabolic pathway	[^11^C]acetate
Amino acids	Radiotracer; increased uptake due to upregulation of transmembrane amino acid transport	[^18^F]fluciclovine
PSMA (Prostate-specific membrane antigen)	Transmembrane protein overexpressed in prostate cancer cells	[^111^In]capromab pendetide *N*-{*N*-[(*S)*-1,3-dicarboxypropyl] carbamoyl},-4-(^18^F)fluorobenzyl-l-cysteine or [^18^F]DCFBC ^68^Ga-*N*,*N*;-bis [2-hydroxy-5-(carboxyethyl)benzyl] ethylenediamine-*N*,*N*′-diacetic acid or [^68^Ga]HBED-CC [^68^Ga]PSMA
uPAR (Urokinase plasminogen activator receptor)	Cell membrane receptor with overexpression in prostate cancer cells	[^68^Ga]DOTA-AE105
